# Partial replacement of soybean meal with mixed plant proteins yields comparable growth and carcass quality in growing-finishing pigs

**DOI:** 10.3389/fvets.2025.1527352

**Published:** 2025-01-22

**Authors:** Wei Han Zhao, Joo Hyun Ha, Sungbo Cho, In Ho Kim

**Affiliations:** ^1^Department of Animal Biotechnology, Dankook University, Cheonan, Republic of Korea; ^2^Smart Animal Bio Institute, Dankook University, Cheonan, Republic of Korea

**Keywords:** plant protein products, growth performance, nutrient digestibility, fecal scores, meat quality

## Abstract

**Objective:**

This study evaluated the impacts of partial replacement of soybean meal with different concentrations of mixed plant protein products (rapeseed meal (RSM) - palm kernel meal (PKM) -distillers dried grains with soluble (DDGS)) on growth performance and carcass quality of growing-finishing pigs.

**Methods:**

A total of 180 crossbred [Yorkshire x Landrace] pigs with average initial weight of 29.72 + 1.65 Kg were randomly assigned to one of five dietary treatment groups on the basis of weight and sex, and the experimental duration was 105 days. The basal diet (C23ON) of growing and finishing pigs were partially replaced with increasing level of RSM-PKM-DDGS (1 to 5% for growing pigs, and 2 to 6% for finishers). Each treatment group had 9 replicate pens, each containing 2 barrows and 2 gilts. During the 15-week trial, body weight (BW), average daily gain (ADG), average daily feed intake (ADFI), and feed conversion ratio (FCR) were calculated for the periods of weeks 0–5, weeks 5–10, week 10–15, and for the entire experimental period.

**Results:**

The partial replacement of soybean meal with mixed plant protein products (RSM, PKM-DDGS) showed no significant effect on the growth performance of pigs during the entire experimental period (*p* > 0.05). However, a decreasing ADG (*p* = 0.0837) and ADFI (*p* = 0.0779) were observed during weeks 0–5, while an increasing FCR was noted during weeks 10–15 (*p* = 0.0835) and the overall period. Furthermore, the replacement of soybean meal with mixed plant protein products (RSM-PKM-DDGS) showed no linear or quadratic effects on the digestibility of dry matter (DM), nitrogen (N), energy (E), fecal scores, or meat quality.

**Conclusion:**

This suggests that mixed plant protein products (RSM, PKM, and DDGS) can effectively replace soybean meal as the primary protein source, providing comparable outcomes while potentially reducing feed costs.

## 1 Introduction

Exploring feed in diets for pigs has garnered significant attention in the livestock industry, for example, soybean meal (SBM) is a major protein source for livestock, but it is also a relatively expensive feed ingredient, and feed costs account for approximately 50% of the total cost in pig production systems (1). Therefore, the partial substitution of feed ingredients to reduce feed costs has become increasingly important, with the aim of achieving higher growth performance at lower production costs (2). Additionally, soybean meal (SBM), another primary feed ingredient, is a byproduct of soybean oil production, Due to its high protein content, well-balanced amino acid profile, and excellent digestibility, SBM is widely used in diets of pigs, serving as a critical protein source in the livestock industry that effectively promotes pig growth and improves feed utilization (3). Moreover, the sharp increase in soybean prices has driven many researchers to seek alternative, cost-effective protein sources (4). Rapeseed meal (RSM) has emerged as a favored protein source in pig’s feed due to its high protein content and lower cost, becoming increasingly popular in pig nutrition (5). However, the high fiber content (10–20%) and lower oligosaccharide levels in RSM result in reduced energy utilization, which limits its efficiency in diets to some extent, thereby potentially reducing overall growth performance (6). Furthermore, some reports indicated that the inclusion of up to 4% RSM in the diets of growing pigs had no adverse effects on growth performance, nutrient digestibility, or meat quality (7). Also important is Palm kernel meal (PKM), a byproduct of palm kernel oil extraction, primarily produced in Southeast Asia, with Malaysia and Indonesia being the largest producers, due to its price advantage, low risk of mycotoxins, and relatively stable quality, PKM has been widely adopted as a substitute for SBM in animal feed (8). Studies have shown that the inclusion of PKM in pig diets can partially replace SBM while promoting growth, nutrient digestibility, and meat quality (9). With the rapid rise of the fuel ethanol industry, distillers dried grains with solubles (DDGS), a primary byproduct of ethanol production, has seen a significant increase in production capacity. During the dry-grind ethanol production process, the starch in grains is converted into ethanol and carbon dioxide, resulting in a concentration of nutrients in DDGS. Compared to original corn, DDGS contains approximately three times the protein, oil, fiber, and mineral content, making it a highly promising protein source for animal feed (10). The use of DDGS, particularly in the early stages of pig feeding, can help substantially reduce feed costs, thereby enhancing profitability while maintaining growth performance (11). Despite its notable nutritional advantages, research has shown that excessive inclusion of DDGS in the diet can negatively affect feed efficiency and lean meat percentage, significantly reducing carcass yield and impacting overall meat quality (12). Therefore, using DDGS as a partial replacement for SBM or combining it with other plant byproducts could improve the nutritional profile of DDGS, enhance growth performance while, allowing for the maintenance of growth performance while minimizing the negative effects in pigs. The objective of this study is to evaluate the effects of partial replacement of soybean meal with different concentrations of a blend of plant protein products (RSM, PKM, and DDGS) on the growth performance and carcass quality of growing-finishing pigs.

## 2 Materials and methods

### 2.1 Ethical statement

The Animal Care and Use Committee of Dankook University approved all experimental protocols used in the DK-1-2305.

### 2.2 Experimental animals, designs, diets and housing

A total of 180 crossbred pigs [(Yorkshire × Landrace) × Duroc], with an average body weight (BW) of 29.72 ± 1.65 kg, were used in a 15-week trial. The pigs were randomly assigned to one of five dietary treatments in a completely randomized block design comprising of nine replicate pens per treatment. Four pigs per pen (2 barrows and 2 gilts) were arranged according to their initial body weight and sex. The dietary treatments were as follows: The basal diets (CON) of growing and finishing pigs were partially replaced with increasing levels of RSM-PKM-DDGS (1 to 5% for growing pigs, and 2 to 6% for finishers). All diets were formulated to meet or exceed the nutritional requirements specified by the National Research Council (13) guidelines (Table 1). Pigs in both experiments were housed in controlled environments with plastic slatted flooring. Each pen was equipped with a self-feeder and a nipple drinker, allowing pigs to have ad libitum access to feed and water throughout the experiment.

**Table 1:** Composition of growing-finishing pig diets.

Item	(Growing phase)					(Finishing phase)				
	1%	2%	3%	4%	5%	2%	3%	4%	5%	6%
Ingredients (%)										
Corn	73.87	71.84	69.79	67.76	65.72	73.75	71.69	69.68	67.67	65.62
Soybean meal	17.50	16.22	14.95	13.66	12.40	15.02	13.76	12.48	11.20	9.92
Rapeseed meal	1.00	2.00	3.00	4.00	5.00	2.00	3.00	4.00	5.00	6.00
Palm kernel meal	1.00	2.00	3.00	4.00	5.00	2.00	3.00	4.00	5.00	6.00
DDGS	1.00	2.00	3.00	4.00	5.00	2.00	3.00	4.00	5.00	6.00
Tallow	2.35	2.69	3.03	3.37	3.71	2.42	2.77	3.10	3.43	3.78
MDCP	1.45	1.40	1.35	1.30	1.25	1.15	1.10	1.00	0.95	0.90
Limestone	0.75	0.76	0.78	0.80	0.80	0.65	0.67	0.72	0.72	0.74
Salt	0.10	0.10	0.10	0.10	0.10	0.10	0.10	0.10	0.10	0.10
Methionine (99%)	0.05	0.05	0.05	0.05	0.05	0.06	0.05	0.05	0.05	0.05
Lysine (78%)	0.50	0.51	0.52	0.53	0.54	0.42	0.43	0.44	0.45	0.46
Mineral mix^1^	0.20	0.20	0.20	0.20	0.20	0.20	0.20	0.20	0.20	0.20
Vitamin mix^2^	0.20	0.20	0.20	0.20	0.20	0.20	0.20	0.20	0.20	0.20
Choline (25%)	0.03	0.03	0.03	0.03	0.03	0.03	0.03	0.03	0.03	0.03
Total	100.00	100.00	100.00	100.00	100.00	100.00	100.00	100.00	100.00	100.00
Calculated value										
CP, %	15.50	15.50	15.50	15.50	15.50	15.00	15.00	15.00	15.00	15.00
ME (kcal/kg)	3,300	3,300	3,300	3,300	3,300	3,300	3,300	3,300	3,300	3,300
Ca, %	0.70	0.70	0.70	0.70	0.70	0.60	0.60	0.60	0.60	0.60
P, %	0.60	0.60	0.60	0.60	0.60	0.55	0.55	0.55	0.55	0.55
Lys, %	1.10	1.10	1.10	1.10	1.10	1.00	1.00	1.00	1.00	1.00
Met, %	0.30	0.30	0.30	0.30	0.30	0.30	0.30	0.30	0.30	0.30
FAT, %	5.25	5.61	5.97	6.33	6.69	5.40	5.76	6.11	6.47	6.83

DDGS, distillers dried grains solubles; MDCP, mono dicalcium phosphate; CP, crude protein; ME, metabolizable energy; LYS, lysine; MET, methionine Provided per kg of complete diet: 16,800 IU vitamin A; 2,400 IU vitamin D_3_; 108 mg vitamin E; 7.2 mg vitamin K; 18 mg Riboflavin; 80.4 mg Niacin; 2.64 mg Thiamine; 45.6 mg D-Pantothenic; 0.06 mg. Cobalamine; 12 mg Cu (as CuSO_4_); 60 mg Zn (as ZnSO_4_); 24 mg Mn (as MnSO_4_); 0.6 mg I (as Ca (IO_3_)_2_); 0.36 mg Se (as Na_2_SeO_3_).

### 2.3 Growth performance

During the 15-week duration of the experiment, the individual body weights (BW) of the pigs were recorded at the start of the trial, and then weighed at weeks 5, 10 and 15 of the experimental trial to estimate the body weight gain and average daily weight gain (ADG) on treatment basis. At the same time, the feed intake and feed leftovers were measured to estimate the average daily feed intake (ADFI) while feed conversion ratio (FCR) was evaluated.

### 2.4 Nutrient digestibility

To estimate the apparent total tract digestibility (ATTD), 0.20% chromium oxide (Cr₂O₃) was added to the diets 7 days prior to fecal collection in weeks 5, 10, and 15. Fecal samples were randomly collected from two pigs (one boar and one sow) per pen and pooled on a pen basis. The fecal samples were stored at −20°C in the laboratory before determining the ATTD for dry matter (DM), crude protein (CP), and gross energy (GE). Before chemical analyses, fecal samples were dehydrated at 70°C for 72 h. Feed and fecal samples were then ground and sieved through a 1 mm sieve to obtain a homogeneous sample. All samples were analyzed for DM, CP, and DE according to the Association of Official Analytical Chemists (14). Chromium concentration in the samples was measured using a UV spectrophotometer (Optizen POP, South Korea) according to (15). Total energy was measured by an oxygen bomb calorimeter (Parr instrument, United States). Nitrogen (N) was analyzed with a Kjeltec 2,300 nitrogen analyzer (Foss Tecator AB, Denmark). The ATTD was estimated using the following formula:


$$\mathrm{ATTD}\;\left(\%\right)=[1-\{\left(N_{f}\times C_{d}\right)/(N_{d}\times C_{f}\}]\times 100$$

where: N_f_ indicated concentration in feces (% DM), N_d_ indicated nutrient concentration in diets (% DM), *C_f_* indicated chromium concentration in feces (% DM), and C_d_ indicated chromium concentration in diets (% DM).

### 2.5 Fecal score

At the start of the experiment, as well as during the 5th, 10th, and 15th weeks, the fecal score was determined by averaging the scores of four pigs in each pen using a 5-grade scoring system. The standard of this system is as follows: 1 = hard, dry pellets in a small, hard mass; 2 = hard, formed stool that remains firm and soft; 3 = soft, formed and moist stool that retains its shape; 4 = soft, unformed stool that assumes the shape of the container; 5 = watery, liquid stool that can be poured. Scores were recorded on a pen basis following observations of individual pigs and signs of stool consistency in the pen, all pigs had mash form of feed.

### 2.6 Back fat thickness and lean meat percent of finishing pigs fed experimental diets

At the start of the second phase, and at the end of weeks 5 and 10, the backfat thickness and Lean Meat Percentage (LMP) of all pigs were measured. A real-time ultrasound instrument (Piglot 105; SFK Technology, Herlev, Denmark) was used to measure the carcass backfat thickness and LMP. LMP was calculated for all pigs (40 per treatment) from three different sites (shoulder, mid-back, and loin, just above the elbow, the last rib and the last lumbar vertebrae, respectively) 5 cm to the right of the midline according to the procedure described by Upadhaya et al. (16).

### 2.7 Meat quality

Carcasses were chilled at 2°C for 24 h and a piece of the right loin was taken through a perpendicular cut between the 10th and 11th ribs. Before evaluating meat quality, meat samples were thawed at ambient temperature. The color measurement of lightness (L*), redness (a*), and yellowness (b*) values were determined with a Minolta CR410 chromameter (Konica Minolta Sensing, Inc., Osaka, Japan). Sensory evaluation (color, marbling, and firmness scores) was carried out according to the National Pork Producers Council standards (17). At the same time, duplicate pH values of each sample were measured with a pH meter (Fisher Scientific, Pittsburgh, PA, United States). The water-holding capacity (WHC) was measured based on the procedure described in a previous report (18). Briefly, a 0.3 g sample was pressed at 3000 psi for 3 min on a 125-mm-diameter filter paper. The areas of the pressed sample and expressed moisture were then determined with a digitizing area-line sensor (MT-10S, M.T. Precision Co. Ltd., Tokyo, Japan). The ratios of water to meat areas were calculated as a measure of WHC (a smaller ratio indicates higher WHC). The longissimus muscle area (LMA) was measured by tracing the longissimus muscle surface at the 10th rib, which also used the above-mentioned digitizing area-line sensor. Then, a 4 g of meat sample was stored in a plastic bag and treated in a water bath (100°C) for 5 min for measuring cooking loss. Then samples were cooled at room temperature. Cooking loss was calculated as:


$$\begin{array}{l} \mathrm{Cooking loss}\\ =\frac{\mathrm{sample weig}ht\;\mathrm{before cooking}-\mathrm{sample weig}ht\;\mathrm{after cooking}}{\mathrm{sample weig}ht\;\mathrm{before cooking}}\;\times 100. \end{array}$$

Drip loss was measured using approximately 4.5 g of meat sample according to the plastic bag method. On days 1, 3, 5, and 7, the meat samples were removed and dried on paper towels, then their weight was checked. Differences between sample weights were used to calculate the drip loss.

### 2.8 Carcass grade

Backfat thickness (BFT) (mm), carcass weight, and carcass grade were assessed. The quality of pork carcasses was graded into “Grade 1+,” “Quality Grade 1,” or “Grade 2,” based on characteristics such as marbling, lean color, and conditions of belly streaks.

### 2.9 Statistical analysis

All data in this experiment were analyzed according to a completely randomized block design using GLM SAS (Statistical Analysis System, Version 9.2); each pen was treated as an experimental unit, except for meat quality, where individual pigs were considered an experimental unit. Duncan’s multiple range test was performed to determine group differences. Orthogonal polynomials were used to evaluate the linear and quadratic effect of increasing (RSM-PKM-DDGS) supplementation to the diet. The initial body weight was utilized as a covariate for ADG and ADFI. Data variability was expressed as SEM, with a *p*-value less than 0.05 considered statistically significant, and a p-value from 0.05 to 0.10 considered a trend.

## 3 Results

The effects of partially replacing soybean meal in diets with different concentrations of a mixed plant protein product (RSM-PKM-DDGS) on the growth performance of growing-finishing pigs are shown in Table 2. Throughout the entire trial period, there were no significant differences (*p* > 0.05) in body weight, ADG (average daily gain), ADFI (average daily feed intake), and FCR (feed conversion ratio) among the experimental groups except for 0 to 5 weeks. During 0 to 5 weeks, The ADG and ADFI of growing-finishing pigs fed group 4 diet were lower compared to those fed the CON group. Additionally, during weeks 0 to 5, the ADG (*p* = 0.0837) and ADFI (*p* = 0.0779) showed a decreasing trend, and during weeks 10–15, FCR (*p* = 0.0835) showed an increasing trend throughout the entire period. The effects of partially replacing soybean meal in diets with different concentrations of a mixed plant protein product (RSM-PKM-DDGS) on the fecal scores of growing-finishing pigs are shown in Table 3. Throughout the entire trial period, there was no significant difference (*p* > 0.05) in fecal scores among the experimental groups. The effects of partially replacing soybean meal in diets with different concentrations of a mixed plant protein product (RSM-PKM-DDGS) on the nutrient digestibility of growing-finishing pigs are shown in Table 4. Throughout the entire trial period, there was no significant difference (*p* > 0.05) in nutrient digestibility among the experimental groups. Throughout the entire trial period, there was no significant difference (*p* > 0.05) in backfat thickness and LMP, and meat quality among the experimental groups (Tables 5, 6). Furthermore, there was no significant difference (p > 0.05) in carcass grade among the experimental groups until week 15 (Table 7).

**Table 2:** Performance of growing-finishing pigs fed different inclusion levels of mixed plant protein products.

Items	CON	TRT1	TRT2	TRT3	TRT4	SEM	*p*- value	
Body weight, kg							Linear	Quadratic
Initial	29.72	29.72	29.72	29.72	29.72	0.003	0.9986	0.9985
Week 5	55.42	54.41	54.09	54.32	53.85	0.45	0.3514	0.4762
Week 10	83.66	83.31	83.35	82.85	83.12	0.56	0.6114	0.9436
Week 15	115.25	114.09	114.20	112.97	113.57	1.25	0.3334	0.9819
Week 0–5								
ADG, g	734^a^	706^ab^	696^ab^	703^ab^	689^b^	13	0.0837	0.1872
ADFI, g	1919^a^	1864^ab^	1845^ab^	1859^ab^	1830^b^	25	0.0779	0.1672
FCR	2.617	2.644	2.652	2.647	2.656	0.014	0.1397	0.6053
Week 5–10								
ADG, g	800	789	790	775	783	17	0.2939	0.8981
ADFI, g	2,201	2,192	2,194	2,178	2,186	27	0.6095	0.9005
FCR	2.753	2.783	2.780	2.815	2.796	0.030	0.1655	0.9171
Week 10–15								
ADG, g	903	879	881	861	870	21	0.1805	0.9545
ADFI, g	2,754	2,712	2,723	2,710	2,713	35	0.4300	0.6709
FCR	3.058	3.095	3.095	3.151	3.126	0.036	0.0835	0.7864
Overall								
ADG, g	847	830	832	815	823	18	0.2161	0.9793
ADFI, g	2,461	2,435	2,443	2,428	2,433	29	0.4785	0.8464
FCR	2.912	2.939	2.940	2.982	2.961	0.300	0.0903	0.7962

ADFI, average daily feed intake; ADG, average daily gain; FCR: feed conversion ratio; SEM: pooled standard error of the mean. Dietary treatments were as follows: (1) CON-basal diet, (RSM-PKM-DDGS) 1% for growing pigs and 2% for finishing pigs; (2) TRT1, (RSM-PKM-DDGS) 2% for growing pigs and 3% for finishing pigs; (3) TRT2, (RSM-PKM-DDGS) 3% for growing pigs and 4% for finishing pigs; (4) TRT3, (RSM-PKM-DDGS) 4% for growing pigs and 5% for finishing pigs; (5) TRT4, (RSM-PKM-DDGS) 5% for growing pigs and 6% for finishing pigs. ^a, b^ means in the row with superscripts denotes statistically significant.

**Table 3:** Fecal scores of growing-finishing pigs fed varied inclusion levels of mixed plant protein products.

Items	CON	TRT1	TRT2	TRT3	TRT4	SEM	p-value	
Fecal score^1^							Linear	Quadratic
Initial	3.34	3.33	3.22	3.26	3.29	0.06	0.2221	0.7109
Week 5	3.13	3.15	3.17	3.16	3.20	0.07	0.6720	0.7477
Week 10	3.15	3.17	3.16	3.21	3.19	0.034	0.2386	0.5856
Week 15	3.07	3.10	3.09	3.12	3.11	0.030	0.3993	0.9113

SEM, pooled standard error of the mean. Dietary treatments were as follows: (1) CON-basal diet, (RSM-PKM-DDGS) 1% for growing pigs and 2% for finishing pigs; (2) TRT1, (RSM-PKM-DDGS) 2% for growing pigs and 3% for finishing pigs; (3) TRT2, (RSM-PKM-DDGS) 3% for growing pigs and 4% for finishing pigs; (4) TRT3, (RSM-PKM-DDGS) 4% for growing pigs and 5% for finishing pigs; (5) TRT4, (RSM-PKM-DDGS) 5% for growing pigs and 6% for finishing pigs; ^1^Fecal score = 1 hard, dry pellet; 2 firm, formed stool; 3 soft, moist stool that retains shape; 4 soft, unformed stool that assumes shape of container; 5 watery liquid that can be poured.

**Table 4:** Nutrient digestibility of growing-finishing pigs fed varied inclusion levels of mixed plant protein products.

Items	CON	TRT1	TRT2	TRT3	TRT4	SEM	*p*-value	
Week 5							Linear	Quadratic
Dry matter	80.68	80.54	80.36	80.42	80.08	0.83	0.7987	0.9006
Crude protein	77.15	76.97	76.71	76.80	76.52	0.89	0.7259	0.8776
Energy	80.99	80.87	80.67	80.75	80.46	0.52	0.7448	0.8760
Week 10								
Dry matter	75.23	74.96	74.99	74.82	74.90	0.91	0.7559	0.9525
Crude protein	71.58	71.41	71.43	71.20	71.28	0.83	0.7549	0.9702
Energy	74.26	74.07	74.10	73.90	73.99	0.81	0.7688	0.9950
Week 10–15								
Dry matter	72.23	71.95	71.97	71.77	71.86	1.01	0.7431	0.9686
Crude protein	69.24	68.97	69.02	69.76	68.85	0.54	0.5583	0.4202
Energy	71.18	70.98	71.01	70.76	70.86	0.71	0.6960	0.9730

SEM, pooled standard error of the mean. Dietary treatments were as follows: (1) CON-basal diet, (RSM-PKM-DDGS) 1% for growing pigs and 2% for finishing pigs; (2) TRT1, (RSM-PKM-DDGS) 2% for growing pigs and 3% for finishing pigs; (3) TRT2, (RSM-PKM-DDGS) 3% for growing pigs and 4% for finishing pigs; (4) TRT3, (RSM-PKM-DDGS) 4% for growing pigs and 5% for finishing pigs; (5) TRT4, (RSM-PKM-DDGS) 5% for growing pigs and 6% for finishing pigs.

**Table 5:** BFT and LMP of growing-finishing pigs fed varied inclusion levels of mixed plant protein products.

Items	CON	TRT1	TRT2	TRT3	TRT4	SEM	*p*- value	
Initial							Linear	Quadratic
Backfat thickness, mm	12.40	12.38	12.36	12.35	12.36	0.02	0.9750	0.9938
LMP, %	62.17	62.18	62.16	62.17	62.18	0.01	0.9985	0.9985
Week 10								
Backfat thickness, mm	15.71	15.42	15.60	15.32	15.51	0.30	0.6861	0.9919
LMP, %	56.74	56.29	56.56	56.18	56.40	0.23	0.1785	0.8890
Week 15								
Backfat thickness, mm	18.96	18.68	18.82	18.57	18.71	0.28	0.6768	0.9835
LMP, %	52.33	51.85	52.10	51.77	51.97	0.23	0.1677	0.7629

LMP, Lean meat percentage; SEM: pooled standard error of the mean. Dietary treatments were as follows: (1) CON-basal diet, (RSM-PKM-DDGS) 1% for growing pigs and 2% for finishing pigs; (2) TRT1, (RSM-PKM-DDGS) 2% for growing pigs and 3% for finishing pigs; (3) TRT2, (RSM-PKM-DDGS) 3% for growing pigs and 4% for finishing pigs; (4) TRT3, (RSM-PKM-DDGS) 4% for growing pigs and 5% for finishing pigs; (5) TRT4, (RSM-PKM-DDGS) 5% for growing pigs and 6% for finishing pigs.

**Table 6:** Meat quality of growing-finishing pigs fed varied inclusion levels of mixed plant protein products.

Items	CON	TRT1	TRT2	TRT3	TRT4	SEM	*p*- value	
Finish							Linear	Quadratic
pH	5.62	5.67	5.66	5.68	5.59	0.05	0.5399	0.7578
Water holding capacity, %	46.32	38.88	40.62	45.98	37.10	3.16	0.9653	0.0958
Longissimus muscle area, cm^2^	8020.89	8081.20	8214.81	8443.65	7944.28	242.45	0.2098	0.7296
Meat color								
L*	54.75	53.59	55.93	52.95	54.89	1.00	0.4681	0.3348
a*	15.17	14.96	14.73	15.35	14.80	0.29	0.8232	0.1569
b*	7.3a	6.17b	7ab	6.32ab	6.77ab	0.36	0.1677	0.5021
Cooking loss, %	27.71	31.34	28.32	29.29	24.87	2.1	0.8566	0.5341
Drip loss, %								
d1	0.74	0.78	0.69	0.82	0.78	0.16	0.8560	0.7952
d3	2.07	2.40	1.84	2.03	2.66	0.31	0.6990	0.8584
d5	3.78	4.08	3.84	3.89	4.11	0.10	0.8473	0.2517
d7	5.87	6.17	5.99	6.01	6.20	0.11	0.5840	0.1904
Sensory evaluation								
Color	3.00	3.00	3.00	3.25	3.00	0.11	0.1544	0.2811
Marbling	3.00	3.17	4.00	2.83	3.08	0.39	0.8365	0.0820
Firmness	3.08	2.75	3.17	2.50	3.42	0.32	0.3431	0.5943

SEM, pooled standard error of the mean. Dietary treatments were as follows: (1) CON-basal diet, (RSM-PKM-DDGS) 1% for growing pigs and 2% for finishing pigs; (2) TRT1, (RSM-PKM-DDGS) 2% for growing pigs and 3% for finishing pigs; (3) TRT2, (RSM-PKM-DDGS) 3% for growing pigs and 4% for finishing pigs; (4) TRT3, (RSM-PKM-DDGS) 4% for growing pigs and 5% for finishing pigs; (5) TRT4, (RSM-PKM-DDGS) 5% for growing pigs and 6% for finishing pigs.

**Table 7:** Carcass grade of growing-finishing pigs fed varied inclusion levels of mixed plant protein products.

Items	CON	TRT1	TRT2	TRT3	TRT4	SEM	*p*- value	
Finish							Linear	Quadratic
Carcass weight, kg	89.89	89.22	89.25	88.97	89.08	0.92	0.5107	0.8335
Backfat thickness, mm	18.78	18.31	18.56	18.14	18.47	0.56	0.4864	0.9586
1+, %	36.11	30.56	33.33	27.78	30.56	–		
1, %	33.33	33.33	36.11	33.33	30.56	–		
2, %	30.56	36.11	30.56	38.89	38.89	–		

SEM, pooled standard error of the mean. Dietary treatments were as follows: (1) CON-basal diet, (RSM-PKM-DDGS) 1% for growing pigs and 2% for finishing pigs; (2) TRT1, (RSM-PKM-DDGS) 2% for growing pigs and 3% for finishing pigs; (3) TRT2, (RSM-PKM-DDGS) 3% for growing pigs and 4% for finishing pigs; (4) TRT3, (RSM-PKM-DDGS) 4% for growing pigs and 5% for finishing pigs; (5) TRT4, (RSM-PKM-DDGS) 5% for growing pigs and 6% for finishing pigs.

## 4 Discussion

In agricultural livestock production, various plant protein products can partially replace soybean meal, such as RSM. While RSM has a high protein content, making it a viable alternative to soybean meal, its high fiber content can impact nutrient digestibility. Therefore, many researchers have aimed to enhance the nutritional digestibility of RSM by improving processing techniques and adding enzymes, such as carbohydrase enzymes, to reduce fiber and anti-nutritional factors in RSM (7). Studies have reported that supplementing with RSM, in contrast to SBM, does not negatively affect body weight, ADG, ADFI, or G/F (19). Which aligns closely with our experimental results, albeit with a slight downward trend in ADG and ADFI during weeks 0 to 5. Evidence from previous research revealed that gradually increasing RSM levels in the diet (2, 4, and 6%) leads to a linear decline in ADG (2). In our study, the decreased ADG was caused by decreased ADFI. Transition from weaning diet (corn-SBM based) to unconventional diet containing RSM-PKM-DDGS can cause initial reduction of feed intake, we believe that the possible reason for this situation could be that RSM, PKM, and DDGS contain higher fiber levels compared to soybean meal, which reduces palatability and increases the gastrointestinal burden, making pigs less willing to consume the feed, thereby reducing feed intake. It is also possible that growing pigs need time to adapt to the texture, taste, and composition of the new diet, as well as to the new environment. During the initial transition phase, the reduction in feed intake is a normal phenomenon as pigs gradually adjust to the new diet and surroundings. It has also been reported that entirely replacing SBM with RSM can result in darker pork, with a significant reduction in meat lightness (L*) and yellowness (b*) values compared to pigs fed SBM. The lower L* values in pork might correlate with reduced fat content (5). These findings differ from our results, possibly due to the relatively low concentration of RSM used in our study. It remains unclear the possible effects of origin and storage temperature of RSM on growth performance of experimental animals, which may account for the absence of significant differences observed in our trial. Thus, further research and detailed analysis are essential. Soybean meal is a more balanced source of essential amino acids, while palm kernel meal (PKM) contains higher levels of methionine but is deficient in some other amino acids, such as threonine, cysteine, and proline. Despite these differences, PKM can still serve as a substitute protein source for corn-soybean meal due to its high protein content and cost-effectiveness, making it a satisfactory alternative (20). Some studies indicated that adding 4% PKM to the diet does not impact carcass characteristics or pork quality in finishing pigs (9), this supports the findings of our study. However, earlier studies show that higher inclusion of PKM in the diet can lead to a linear decline in ADFI, crude protein, and crude fiber digestibility, Pigs fed a PKM-based diet exhibit slower growth rates, poorer feed conversion ratios, and reduced feed intake (21). Additionally, research has shown that including 40% PKM in the diet can lower feed costs for weaning piglets; however, PKM should not exceed 35% in the diet of growing pigs as it may significantly impact body weight (22). This is in contrast with the results of our findings, because the PKM was mixed with RSM and DDGS at relatively low inclusion levels, which mitigated any adverse effects. DDGS, a by-product of the bioethanol industry after ethanol extraction, is highly regarded as livestock feed due to its rich nutritional profile and cost-effectiveness. DDGS is abundant in crude protein, fats, and dietary fiber, and contains substantial amino acids, vitamins, and minerals. As a non-conventional feed ingredient, because DDGS contains lower levels of antioxidants and anti-nutritional factors, along with higher nutritional value, it can serve as an alternative protein source to soybean meal. This versatility has led to its widespread application in feed for swine, poultry, and ruminants (23). Studies indicate that incorporating 4–15% DDGS into diets generally does not adversely affect intake, ADG, or G/F in growing pigs (10). Another study noted that supplementing up to 25% DDGS in weaned piglet diets does not impact their overall growth performance (24), which aligns well with our experimental findings. However, other studies have reported that adding 15 and 30% DDGS to a SBM-based diet significantly reduces the ADG and ADFI of nursery pigs throughout the trial, although there is an upward trend in G/F (25). This discrepancy with our results may be due to variability in the nutritional composition of DDGS, which can vary with raw material quality and processing methods. Thus, in practical applications, it is essential to consider the source, batch variations, and inclusion levels of DDGS.

## 5 Conclusion

Our study demonstrates that partial replacement of soybean meal with varying concentrations of mixed plant protein products (RSM-PKM-DDGS) does not negatively impact growth performance, nutrient digestibility, fecal scoring, or meat quality in growing-finishing pigs. At the same time RSM, PKM, and DDGS are nutrient-dense, cost-effective, and widely used potential alternatives. The incorporation of mixed plant protein products (RSM-PKM-DDGS) around 1 to 5% in growing and 2 to 6% in finishing would be suitable levels to improve the performance and to reduce feed cost for sustainable pig production.

## Data Availability

The raw data supporting the conclusions of this article will be made available by the authors, without undue reservation.
